# Mitochondria-Shaping Proteins and Chemotherapy

**DOI:** 10.3389/fonc.2021.769036

**Published:** 2021-11-18

**Authors:** Longlong Xie, Tiansheng Zhou, Yujun Xie, Ann M. Bode, Ya Cao

**Affiliations:** ^1^ Hunan Children’s Hospital, The Pediatric Academy of University of South China, Changsha, China; ^2^ Key Laboratory of Carcinogenesis and Invasion, Chinese Ministry of Education, Department of Radiology, Xiangya Hospital, Central South University, Changsha, China; ^3^ Cancer Research Institute and School of Basic Medical Science, Xiangya School of Medicine, Central South University, Changsha, China; ^4^ The Hormel Institute, University of Minnesota, Austin, MN, United States; ^5^ Research Center for Technologies of Nucleic Acid-Based Diagnostics and Therapeutics Hunan Province, Changsha, China; ^6^ Molecular Imaging Research Center of Central South University, Changsha, China; ^7^ National Joint Engineering Research Center for Genetic Diagnostics of Infectious Diseases and Cancer, Changsha, China

**Keywords:** Mitochondria-shaping proteins, chemotherapy, virus, energy metabolism, targeted drugs

## Abstract

The emergence, in recent decades, of an entirely new area of “Mitochondrial dynamics”, which consists principally of fission and fusion, reflects the recognition that mitochondria play a significant role in human tumorigenesis and response to therapeutics. Proteins that determine mitochondrial dynamics are referred to as “shaping proteins”. Marked heterogeneity has been observed in the response of tumor cells to chemotherapy, which is associated with imbalances in mitochondrial dynamics and function leading to adaptive and acquired resistance to chemotherapeutic agents. Therefore, targeting mitochondria-shaping proteins may prove to be a promising approach to treat chemotherapy resistant cancers. In this review, we summarize the alterations of mitochondrial dynamics in chemotherapeutic processing and the antitumor mechanisms by which chemotherapy drugs synergize with mitochondria-shaping proteins. These might shed light on new biomarkers for better prediction of cancer chemosensitivity and contribute to the exploitation of potent therapeutic strategies for the clinical treatment of cancers.

## Introduction

Mitochondria are important dynamic organelles which can remodel morphology and functions, when cells are exposed to severe conditions, such as hypoxia, viral infections, and nutrient deprivation (1, 2). These cellular organelles are directly involved in the development of diseases such as diabetes, neuropathy, cardiovascular malfunctions, and cancer (3, 4). Mitochondrial dynamics consist principally of two mutually constrained remodeling processes, mitochondrial fission and fusion (5). Several shaping proteins are involved in this process and mainly include fission proteins, such as mitochondrial dynamin-related protein 1 (Drp1) and mitochondrial outer membrane receptor proteins [i.e., mitochondrial fission 1 protein (Fis1), mitochondrial fission factor (Mff), and mitochondrial dynamics protein of 49/51kDa (MiD49/51)], and fusion proteins such as mitofusin1/2 (Mfn1/2) and optic atrophy 1 (OPA1) (6, 7). Tumor cells can adjust their mitochondrial morphology in response to specific stressors to maintain functions that can promote tumor phenotypes (8). These adjustments have pivotal significance in tumorigenesis, ranging from enhanced malignant transformation and tumor progression to the impact on the response to treatment and anticancer immune monitoring (9–12). Importantly, mitochondria are major organelles associated with chemotherapeutic drug resistance and imbalances in mitochondrial dynamics influences sensitivity to chemotherapy, which are related to oxidative stress states, changes in mitochondrial metabolism-related enzymes and metabolites, and alterations in the mitochondrial-associated death pathway (13–15).

## Mitochondrial Dynamics and Cancers

### Mitochondrial Fission and Tumors

**Figure 1:**
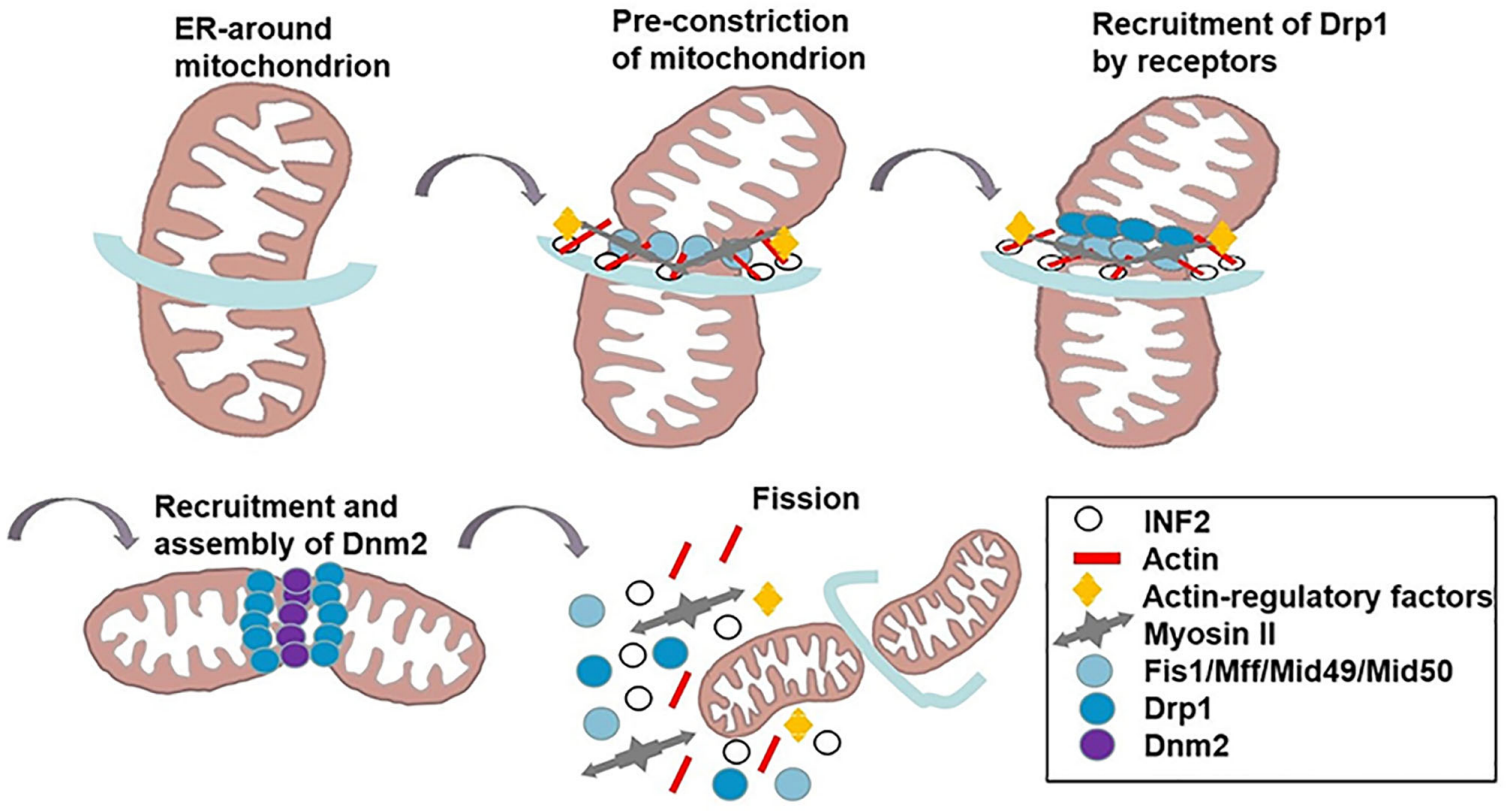
The mechanism of mitochondrial fission. INF2 causes the polymerization of actin at the ER-mitochondrial contact point to generate forces that drive ER tubules around the mitochondria and reduce mitochondrial. Actin-regulatory factors induce actin filament nonequilibrium assembly. Myosin II is recruited to the fission site, causing mitochondrion shrinkage. Subsequently, DRP1 is recruited to the marked division sites to bind to its OMM receptors (Fis 1, Mff, MiD49/51). DNM2 assembles in the DRP1-regulated mitochondrial constriction neck to push mitochondrial scission. GTP hydrolysis results in conformational changes which further augment membrane contraction. Finally, mitochondrion splits to two daughter mitochondria, and the fission complex are disassembled.

Divided mitochondria exhibit dot-like and fragmentary features. This dynamic process participates in tumor heterogeneity, promotes the malignant phenotype, accelerates tumor progression and invasion, and affects treatment and prognosis (16, 17). Mitochondrial fission is thought to be a multi-step and complex process (**Table 1**). Raised cytosolic calcium triggers the activation of endoplasmic reticulum (ER) protein inversion formulator 2 (INF2) which redistributes actin filaments around mitochondria and expands the ER-mitochondria contact point to generate forces that reduce mitochondrial diameter (18, 19, 33); Spire (22), profilin, cofilin, and Arp2/3 (23) are best known as regulators of actin filament, bind to almost all formins (6) that facilitate the actin assembly (34). Then, Myosin II is recruited to the fission site and acts on anti-parallel actin filaments, causing network deformation and leading to mitochondrion shrinkage (20). Drp1, a marker of mitochondrial dynamics, encoded by the *DNM1L* gene, is a cytosolic GTPase (25). The activity of Drp1 is regulated by post-translational modifications, including phosphorylation, ubiquitination, and SUMOylation. Nextly, activated Drp1 is recruited by mitochondrial fission factors like Fis 1, Mff and MiD49/51 to the marked division sites. This binding promotes Drp1 oligomerization, constituting a ring-like structure that benefits an further narrowing of the mitochondrial outer membrane (OMM) (20). Additionally, mitochondrial calcium uniporter (MCU), anchored in the inner mitochondrial membrane (IMM), is the most significant single-way channel responsible for Ca2+ influx into mitochondria (24). MCU upregulates the expression of Drp1/Fis1 and facilitates the migration of Drp1 into mitochondria (35). At the same time, GTP hydrolysis results in conformational changes which further augment membrane contraction. In spite of the fact that Drp1 can make the membrane tubular, it fails to perform membrane cleavage (36). In fact, the GTPase Dynamin 2 (DNM2) assembles in the Drp1-regulated mitochondrial constriction neck to push mitochondrial scission by the adaptor proteins Endophilin B1 (Bif-1 and SH3GLB1) (21, 37). Finally, the mitochondrion splits into two daughter mitochondria and the fission complex is disassembled (**Figure 1**). Generally, phosphorylated Ser616/Ser637 in human and Ser600/Ser579 in mouse levels are critical for the activity of Drp1 (38). Phosphorylation of Drp1 Ser616 promotes the translocation of Drp1 from the cytoplasm to the outer mitochondrial membrane, which upregulates the Drp1 activity, whereas phosphorylation of Ser637 has opposite effects (39, 40). However, a new study revealed that phosphorylated Drp1 Ser637 distributes to both the cytosol and mitochondria. MiD49/51 and Mff interact with phospho-Drp1 Ser637 and nonphospho-Drp1Ser637, which do not play a major role in controlling mitochondrial fission in 293T cells. Importantly, elevated Drp1 activity not only promotes tumor cell proliferation and migration, but also assists in maintaining cell stemness and influences tumor invasion and metastasis as well as response to tumor therapy (41). Recently, inhibition of Drp1 activity in lung cancer cells was reported to promote cancer cell cycle arrest and increase transient apoptosis (42). In addition, analysis of clinical glioma tissue samples revealed that patients with higher levels of phosphorylated Drp1 Ser616 had a poorer survival prognosis (43). These studies further suggest that targeting the Drp1 protein to suppress mitochondrial fission may be a new strategy to overcome tumor survival.

**Table 1:** The functions of mitochondria-shaping proteins.

Mitochondria-shaping proteins	Function	Mechanism	localization	Refs
IFN2	Fission	INF2 response to Ca2+, redistributing actin filaments	ER	(18, 19)
Actin	Fission	Surrounding mitochondria and expands the ER-mitochondron contact sites	Cytosolic	(20, 21)
Myosin II	Fission	Attaches to the fission site, causing actin filaments deformation and shrinkage of mitochondrion	Cytosolic	(20)
Spire	Fission	promotes the actin assembly	Cell nucleus	(22)
Profilin	Fission	Binds to formins and facilitates the actin assembly	Cytosolic	(23)
Cofilin	Fission	Binds to formins and facilitates the actin assembly	Cytosolic	(23)
Arp2/3	Fission	Binds to formins and facilitates the actin assembly	Cytosolic	(23)
MCU	Fission	Responsible for Ca2+ influx into mitochondria and facilitates mitochondrial fission	IMM	(24)
Drp1	Fission	Binding to mitochondrial outer membrane receptor proteins and mediating the formation of mitochondrial fragments	Mainly cytosolic but translocated to the OMM during activation	(21, 25)
Dnm2	Fission	Recruitment by mitochondrial adaptor proteins and constricted mitochondrial neck	Mainly cytosolic but translocated to the OMM during activation	(21)
Fis 1	Fission	Recruitment of Drp1	OMM	(6, 7)
Mff	Fission	Recruitment of Drp1	OMM	(6, 7)
MiD 49/51	Fission	Recruitment of Drp1	OMM	(6)
Endophilin B1	Fission	Recruitment of Dnm2	OMM	(21)
MTP18	Fission	Leads to mitochondrial fission	IMM	(26)
Mfn1	Fusion	OMMs aggregated together	OMM	(27, 28)
Mfn2	Fusion	OMMs aggregated together	OMM	(27, 28)
MSTO1	Fusion	Augments or starts MOMs fusion by interacting with mitofusins	Cytosolic	(29)
MitoPLD	Fusion	Converts cardiolipin to phosphatidic acid and promotes OMMS fusion	OMM	(19)
Bax, Bak	Fusion	Regulate the activity of Mfn2	OMM	(30)
Opa1	Fusion	IMMs fusion	IMM	(31)
Oma1	Fusion	Produces the soluble form of S-Opa1 by cleavaging L-OPA1	IMM	(32)
Yme1l	Fusion	Maintains mitochondrial morphology and complex respiratory activity	IMM	(27)

### Mitochondrial Fusion and Tumors

**Figure 2:**
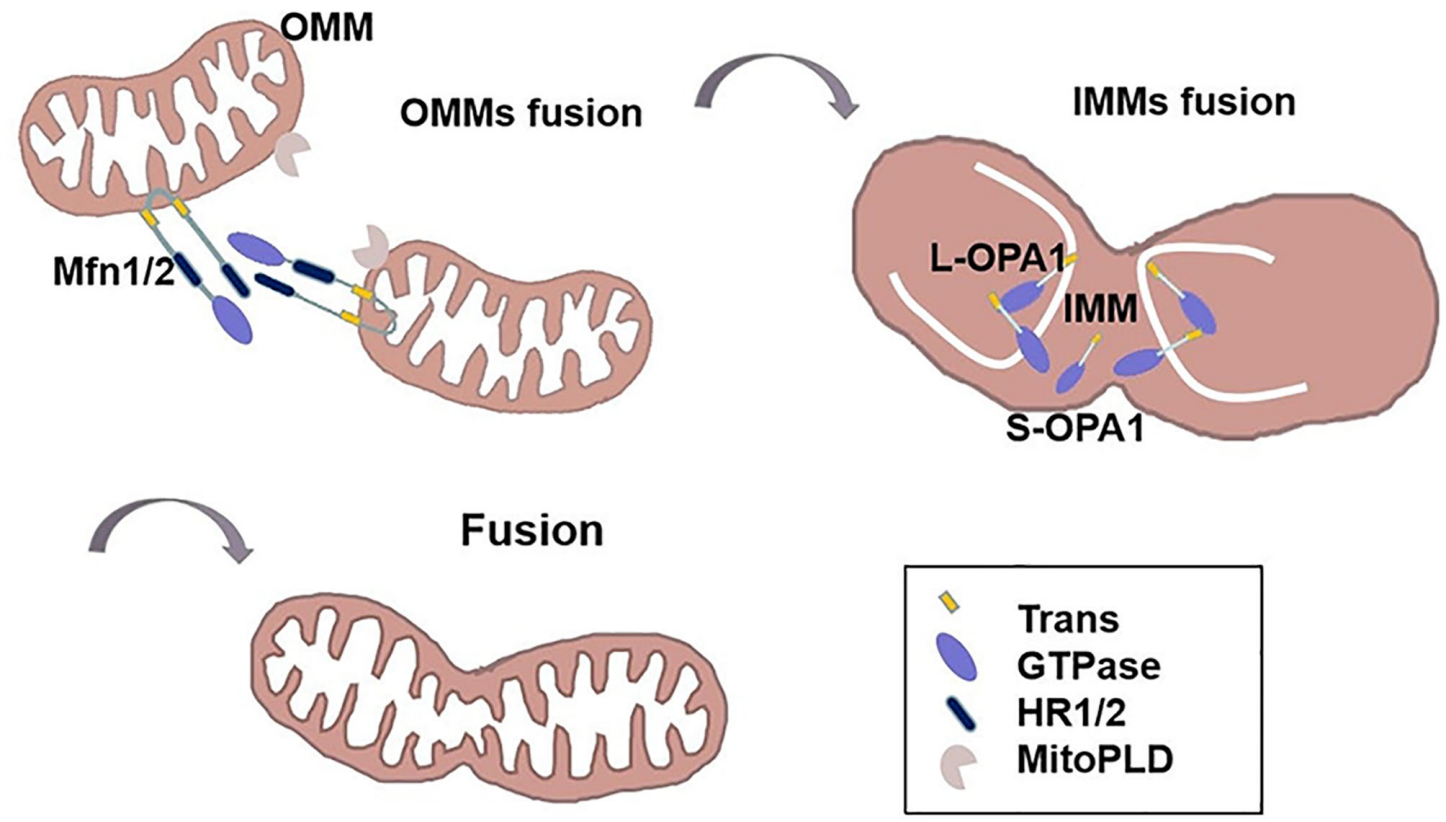
The mechanism of mitochondrial fusion. The HR2 domain of Mfn1/2 constitute an anti-parallel coiled coil and ties the OMMs together in trans. MitoPLD can bring the membranes closer together. Then, GTP hydrolysis triggers a massive conformational rearrangement of Mitofusin, placing two OMMs together. Following outer membrane fusion, L-OPA1 is anchored to the mitochondrial inner membrane, transient head-to-tail assembly of L-OPA1 induces membrane curvature that producing unstable tips on two opposing IMMs. After GTP of L-OPA1 hydrolysis, IMMS fused together. S-OPA1 is located at the mitochondrial intermembrane space.

Fused mitochondria exhibit an interconnected and networked structure. Mitochondrial over-fusion is associated with cancer biology and etiology (27, 44). This dynamic procedure is performed by both outer and inner membranes, with outer membrane fusion mediated by the outer membrane protein, Mfn1/2, and inner membrane fusion mediated by the optic nerve atrophy protein, OPA1 (28, 31) (**Table 1**). Mitofusins contain two transmembrane (trans) domains in between HR1 and HR2 domains. Firstly, the HR2 domain of mitofusins constitute an anti-parallel coiled coil and tie the OMMs together in trans (45). MitoPLD, a member of the phospholipase D family, is bound to the OMM, where it converts cardiolipin to phosphatidic acid, allowing the recruitment of adaptor proteins, bring the membranes closer together (19). Then, GTP hydrolysis triggers a massive conformational rearrangement of mitofusins, which result in mitochondrial pairing and an increase of OMMs junctions (46). In addition, Bax and Bak proteins are involved in regulating the activity of Mfn2. Bax drives the focal localization of Mfn2 on the outer mitochondrial membrane, reduces the membrane mobility and increases the assembly of Mfn2 (30). Moreover, misato (MSTO1) is a soluble cytoplasmic protein that moves to the outer face of the MOM, where it can augment or start MOMs fusion by interacting with mitofusins (29). Following outer membrane fusion, OPA1 is proteolytically hydrolyzed by two endosomal peptidases: Oma1 and the i-AAA protease Yme1l to create two active forms. Long L-OPA1, which is anchored to the mitochondrial inner membrane, and soluble short S-OPA1, which is located at the mitochondrial intermembrane space (32). IMMs fusion is completed by the combined action of L-OPA1 and S-OPA1 (47). Transient head-to-tail assembly of L-OPA1 induces membrane curvature that producing unstable tips on two opposing IMMs (27). After GTP of L-OPA1 hydrolysis, IMMS fused together and cristae maintenance (47) (**Figure 2**). The involvement of Mfn1/2 in the regulation of tumorigenesis is assumed and its activity is less well-reported. Accumulated evidence has shown that tumor cells usually have a low fusion protein phenotype (48–51). For example, lung cancer (49) and colon cancer cells (42) have been shown to often exhibit an imbalance in mitochondrial network structure (i.e., more fission than fusion). This phenotype can be reversed by upregulation of Mfn2, thereby promoting cell cycle arrest and increased apoptosis. Clinically, the Mfn2 protein is poorly expressed in human gastric tumors, with predominant mitochondrial hyper-division and a poor survival prognosis (51). Consistent with this report, lower Mfn2 expression is correlated with more malignant breast tumors (52), which is proposing Mfn1/2 as a tumor suppressor. Moreover, breast cancer cells with low Mfn1 expression are more migratory and thus overexpression of Mfn1 is associated with mitochondrial elongation, which significantly inhibits the metastatic ability of breast cancer cells (53). Recently, cumulative studies have revealed the function of OPA1 in tumor advancement (47, 50, 54). Emerging evidence indicates that hepatocellular carcinomas have higher levels of OPA1 expression compared to non-tumor tissues. Targeting OPA1 depresses mitochondrial fusion and leads to cell death (50). The tubular network of mitochondria meditated by OPA1 favors tumor cell proliferation signals that may be linked to c-Myc activation (54).

## The Mechanism of Mitochondria-Shaping Proteins and Chemotherapy

Mitochondrial dynamics confer bioenergetic plasticity to tumor cells, allowing them to escape chemotherapy-induced death pathways under stressful conditions. Understanding whether mitochondrial fission or fusion serve as pro- or anti-cell death factors is important, specifically with relevance to cell type, cell state, and death initiators (55). Therefore, comprehending the mechanisms of imbalanced mitochondrial dynamics during tumor development and progression is critical for effective cancer treatment.

### Mitophagy Serves as a Bridge to Mitochondria-Shaping Proteins Dependent on Chemosensitivity

Mitophagy is the process of selective mitochondrial degradation through autophagy, an evolutionarily conserved cellular procedure for removing overabundant and impaired mitochondria from eukaryotic organisms (56). The general pathways of mitophagy can be categorized into two forms: ubiquitin-mediated mitophagy and receptor-mediated mitophagy. Parkin can ubiquitinate several outer mitochondrial membrane proteins, such as Mfn1, Mfn2, VDAC (57). Ubiquitinated proteins such as Mfn1/2 can be recognized and bound by autophagy-associated protein LC3 to induce mitochondrial autophagy (58). In the face of stress, mitophagy receptors such as FUNDC1 and BNIP3 can promote mitochondrial fission (59). For example, FUNDC1 acts directly with Drp1 and recruits it to the outer mitochondrial membrane to contribute to mitochondrial fragmentation (60). In addition, BNIP3 can directly interact with OPA1 and facilitate the breakdown of OPA1 oligomers, which increases mitochondrial fission in HeLa cells (61). In the initial stage of chemotherapeutic drug intervention, mitophagy keeps normal cellular metabolism and suppresses tumorigenesis. With the prolongation of chemothrapy, however, the occurrence of mitophagy improves the tolerance of tumor cells and continuously screens tumor cells to retain cells with stemness, leading to chemoresistance (62).

Interestingly, several mitophagy receptors, including BNIP3, NIX and BCL2L13, belong to the BCL2 family (60). BCL2 family proteins have been described to be involved in both apoptosis and mitophagy processes, which keeps them at the center of mitochondrial homeostasis (63). Dysregulation of these key molecules involved in the induction of mitochondrial apoptosis and mitophagy are the main mechanisms of increased tumor chemoresistance, which includes decreases in the ratio of the pro-apoptotic protein, Bax, to the anti-apoptotic protein, Bcl2 (64). Importantly, fragmented mitochondria are conducive to mitophagy (62, 65). Hepatocellular carcinoma (HCC) cells carry their own defense mechanisms, including autophagy and mitophagy. With cisplatin intervention, the activation of Drp1 induced mitophagy in favor of HCC survival. Thus, the Drp1-specific inhibitor, Mdivi-1, targets mitochondrial autophagy, upregulates Bax and downregulates Bcl-xL, increases mitochondrial membrane permeability, and stimulates cytochrome c release, thereby increasing cisplatin-induced apoptosis in HCC (66). Similar with it, a finding in colorectal cancer reported that high-mobility group box 1 protein (HMGB1) secreted from tumor cells after chemoradiotherapy which promotes tumor cells regrowth, proliferation and metastasis. HMGB1/receptor for advanced glycation end product (RAGE)/Erks signal triggers the activation of Drp1, inducing LC3 and p62-dependent mitophagy for chemoresistance in colorectal cancer cells. Furthermore, rectal cancer patients with high phospho-Drp1Ser616 are associated with high risk on developing tumor relapse and poor survival time after chemoradiotherapy treatment (67). These indicate that signals facilitating mitophagy are associated with mitochondrial fission-regulated chemoresistance, whereas apoptosis is often accompanied by mitochondrial fusion-dependent chemosensitivity (68).

Although only limited evidence is available, mitochondrial fission appears to be one of the mechanisms by which cisplatin induces cytotoxicity. Stress-inducible cellular protein P62 acts as a signaling center to modulate multiple cellular traits, such as autophagy and apoptosis. The Bcl2 inhibitor ABT737 triggers selective aggregation of p62 during mitochondrial fission. Increasing the ratio of the Drp1 60kD form to the Drp1 80kD form subsequently activates mitochondria-dependent autophagy, which increases sensitivity to cisplatin. This investigation demonstrates that the 60kD form of Drp1, located in the mitochondria, may be the main pro-fission driver, which could enhance autophagy in favor of chemotherapy (69). However, another study showed that inhibiting mitophagy plays antitumor effects in breast cancer. Liensinine, a new inhibitor of mitophagy, in combination with doxorubicin inhibits the over-accumulation of mitophagosomes to enhance mitochondrial fission-dependent apoptosis and improve chemosensitivity (70).

### P53 in the Mitochondrial Shaping Protein-Regulated Chemotherapy

The C-terminus of the p53 protein contains the mitochondrial localization sequence (71) that enhances mitochondrial localization to mediate chemoresistance. P53 can elevate the activity of Drp1 by its mitochondrial translocation and the phosphorylation of Drp1 Ser616 (72). Cyclooxygenase-2 (COX-2) increases the stemness of nasopharyngeal carcinoma (NPC) cells by enhancing the mitochondrial translocation of p53 that triggers the activation of Drp1. Importantly, the natural compound resveratrol (RSV) inhibits the COX-2/p53/Drp1 signaling axis to reduce mitochondrial fission, leading to increased sensitivity of NPC to the chemotherapy drug, 5-fluorouracil (5-FU) (41). However, a study revealed that p53 is the downstream regulator of Drp1. Repeated administration of nedaplatin contributes to extensive hepatocellular injury and resistance in patients of hepatocellular carcinoma by inactivating the p53/Bcl-2 pathway (73). Additionally, Drp1 has been reported to inhibit the expression of p53 and enhance progression into the HCC cells cycle. Therefore, we demonstrated that mitochondrial shaping protein Drp1 negatively regulates p53 leading to chemoresistance (74).

**Figure 3:**
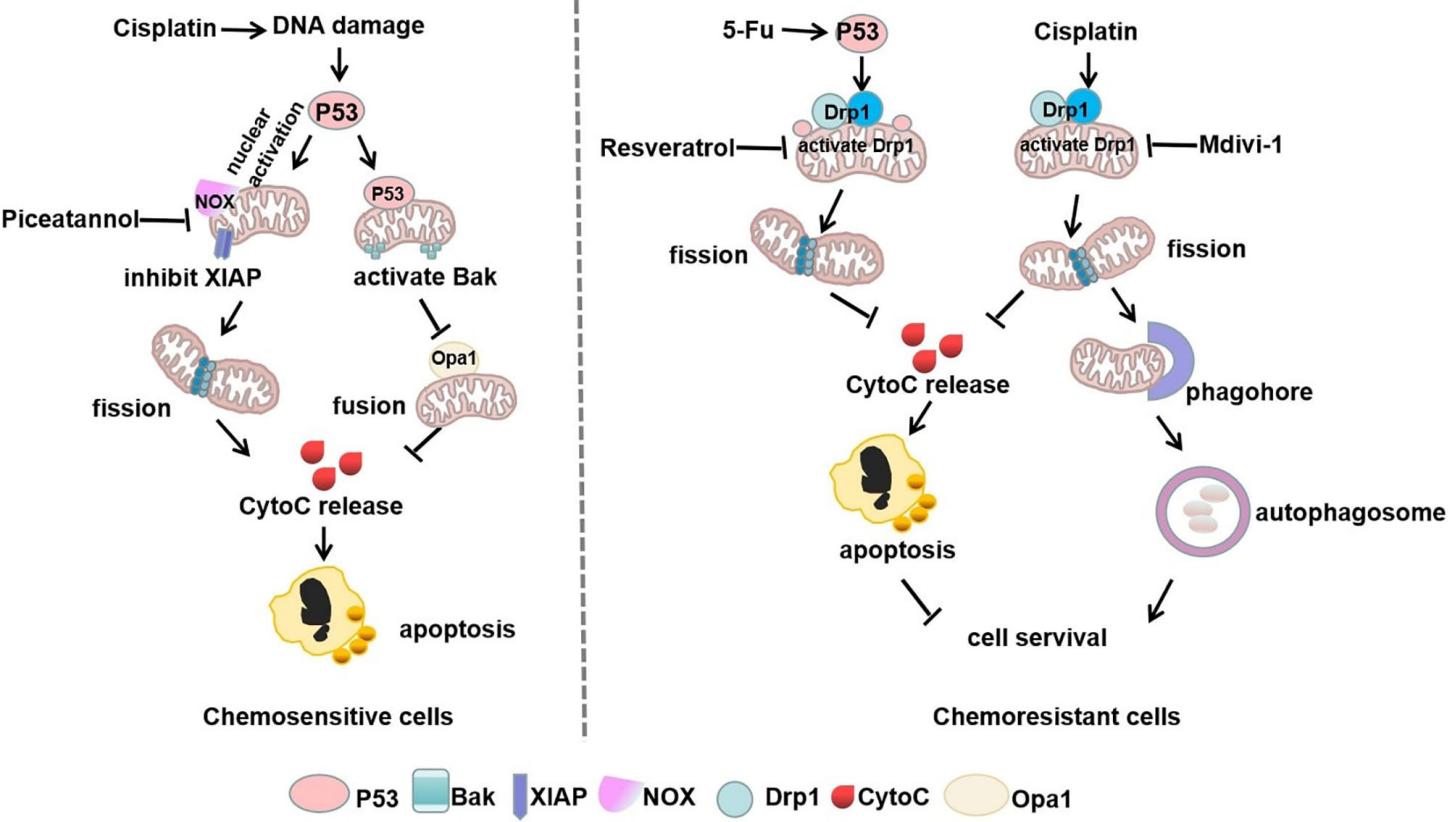
Mitophagy and p53 in mitochondrial shaping protein-regulated chemotherapy. In chemosensitive cells, cisplatin-induced DNA damage exerts dual functions of p53 nuclear activation and mitochondrial translocation, which drives mitochondria-shaping proteins to promote mitochondrial fission and inhibit fusion, thereby increasing apoptosis. However, chemotherapeutic drugs can stimulate p53 mitochondrial translocation or directly increase mitochondrial fission by activation of Drp1, thus promoting autophagy to escape apoptosis in chemoresistant cells.

In addition, p53 exerts the dual function of mitochondrial translocation and nuclear activation, which increases chemotherapy sensitivity by driving mitochondria-shaping proteins. For instance, wild-type p53 moves to the mitochondria in response to cell cycle arrest, DNA damage response, and the induction of apoptosis (75). In gynecological cancers, decreasing the level of phosphorylated Drp1 Ser637 promotes mitochondrial fragmentation and diminishes chemoresistance (76, 77). Piceatannol, an extract from grapes and red wine, is capable of inducing p53 nuclear activation and suppresses the X-linked inhibitor of apoptosis protein (XIAP) (76). Also, the enhanced mitochondrial fission or reduced fusion have been shown to be related to chemoresensitive. Following the cisplatin-induced DNA damage response, p53 is phosphorylated and translocated to the mitochondria, where it pushes the self-cleavage and activation of the mitochondrial protease OMA1. OMA1 inactivates OPA1 by cleaving L-OPA1 to S-OPA1, which inhibits mitochondrial fusion and induces apoptosis (78, 79). Furthermore, mitochondrial chaperone protein CLPB sustains the mitochondrial cristae structure by interacting with the cristae-shaping protein OPA1, while its deletion facilitates apoptosis by causing cristae remodeling and mitochondrial stress reactions. Targeting CLPB conquers venetoclax resistance regulated by p53 loss and renders acute myeloid leukemia (AML) cells sensitive to the co-treatment with Venetoclax and Azacitidine through the induction of pro-apoptotic proteins (80). These studies further indicated that chemotherapy-sensitive cancer cells are mitochondria fission-dependent (**Figure 3**).

### Hypoxia-Induced ROS Stress-Dependent Drp1 Mediated-Mitochondrial Fission in Chemotherapy

Chemotherapy has long been the cornerstone of cancer treatment, but its ability to kill tumor cells is oxygen-dependent. Hypoxia induces an increased expression of Drp1, thereby promoting mitochondrial fission and enhancing the ability of metastatic tumor cells to invade and metastasize in breast cancer. Thus inhibition of Drp1-dependent mitochondrial fragmentation attenuated this hypoxia-induced invasive metastasis (81). Interestingly, this study also reported that cisplatin induced significant apoptosis in cells exhibiting a high mitochondrial fission state. Moreover, Mdivi-1 targeted Drp1 and siRNA silencing of Drp1 effectively increased the mitochondrial membrane potential (MMP), the generation of ROS, and apoptosis in breast cancer cells (81). This suggests that the inherent metastatic properties of breast cancer may be due to the fission of the mitochondria under hypoxia. Drp1-driven mitochondrial division can increase the antitumor activity of cisplatin in breast cancer cells. In contrast, a new reports identified that hypoxia-induced ROS triggers mitochondrial fission by down-regulating phosphorylated Drp1 (Ser637) and Mfn1 expression levels in ovarian cancer cells, thereby inducing cisplatin resistance (82). This may be attributed to the observation that chemoresistant cells showed greater oxidative stress activity and increased mitochondrial division compared to chemosensitive cells, favoring cell proliferation and autophagy (83).

Generally, oncogenic transformation is followed with an increase in ROS levels in cells, contributing to redox imbalance (84). Most tumor cells are killed by oxidized free radicals and excess ROS (85). For example, the inhibitor of *proviral integration site for moloney murine leukemia virus* (PIM) kinases induces the production of mitochondrial ROS by leading to Drp1-dependent mitochondrial fission which results in docetaxel sensitivity (86). Therefore, overcoming anticancer activity and improving the sensitivity of chemotherapy by effectively interfering with hypoxia or ROS is crucial. Notably, oxidative stress is an important mechanism of cisplatin toxicity and mitochondria are the main targets of cisplatin-induced oxidative stress, with excess ROS production eventually leading to cell death. For example, co-culture of bone marrow mesenchymal stem cells (MSCs) with acute leukemia cells stimulates activation of extracellular signal-regulated kinases (ERKs), which trigger Drp1-dependent mitochondrial fragmentation and leads to decreased mitochondrial ROS levels and promotion of the glycolytic phenotypic switch resulting in chemoresistance (87). This study proposes that mitochondria fission interferes with cisplatin-induced oxidative stress to reduce cytotoxicity and escape death, while undergoing metabolic reprogramming to increase metabolic activity, leading to chemoresistance.

## Remodeling of Mitochondria-Shaping Proteins and Energy Metabolism to Modulate Chemoresistance

Tumor metabolism is inextricably linked to mitochondrial dynamics, and mitochondrial fission and fusion are adaptive processes that continually adjust mitochondrial size, shape, and subcellular location to adapt to changes in the cellular environment. These changes obviously contribute to mitochondrial quality control and cellular responses to energy stresses (88).

### Mitochondrial Fission Prefers Glycolysis to Resist Chemotherapy

During metabolic reprogramming of tumor cells, mitochondrial morphology changes, with a predominant preference for fragmentation. Reports indicate that most tumor stem cells are more prone to glycolysis, and such tumors were recognized as glycolysis-addictive tumors, based on increased glucose uptake, lactate production, and expression of glycolytic enzymes, which may be related to their mitochondrial hyper-fragmented state (89–91). Glycolysis-additive cancers include lung, gastric, breast, glioma, colon, neuroblastoma, ovarian, pancreatic, and melanoma with high levels of Drp1 and low levels of Mfn1/2 proteins. Thus, inhibition of Drp1 or overexpression of Mfn1/2 that remodels mitochondrial shape and metabolism could result in decreased proliferation and increased apoptosis of tumor cells (42, 51, 53, 92).

For instance, mitochondrial shaping protein Drp1 affects the metabolic reprogramming of brain tumor-initiating cells (BTICs). Compared with normal neural stem cells, the mitochondria of BTICs are highly fragmented and upregulation of GLUT3 increases glycolytic flux, indicating that the coupling of glycolysis and mitochondrial division is essential in neuronal CSCs (43). Indeed, some findings indicated that oncogene-mediated Drp1 activity-driven metabolic changes are associated with high levels of ROS and glycolytic flux (88, 93, 94). These studies suggest that Drp1-dependent mitochondrial fission and glycolytic metabolism are mutually reinforcing processes in tumor progression and that oncogenic gene-regulated metabolic reprogramming will result in changes in mitochondrial morphology to support metabolic alterations. In addition, deletion of Mfn1 increased a shift in the metabolic pattern of hepatoma cells from OXPHOS to glycolysis, with enhanced cell proliferation and epithelial-mesenchymal transition (EMT) capacity. The glycolysis inhibitor 2-deoxy-glucose (2-DG) reverses vascular invasive metastasis in cancer cells with loss of Mfn1 (95).

These findings imply that mitochondrial fission supports metabolic alterations as a non-negligible factor in tumor initiation and progression. Importantly, it is also closely related to chemotherapy. Some interesting researches revealed that anti-apoptotic protein complex induces mitochondrial fragmentation by up-regulating the translocation of Drp1 to mitochondria and inhibits mitochondrial respiratory complex I, thereby preventing the accumulation of ROS (96, 97). The loss of energy production due to OXPHOS can be compensated for by an increase in glycolysis. Thus, the glycolysis inhibitor 2-DG can attenuate the anti-apoptotic effects of survivin and decrease tumor cell proliferation, making the tumor sensitive to chemotherapeutic agents (96, 97). Recently, we revealed that Epstein-Barr virus latent membrane protein 1 (EBV-LMP1) increases the mitochondrial fission-induced glycolytic metabolic phenotype for NPC cells survival to resist chemotherapy, and phosphorylation of Drp1 Ser616 or dephosphorylation of Drp1Ser637 is essential for LMP1-regulated enhancement of glycolytic metabolism (98). In addition, our earlier studies have shown that LMP1 activates HK2, a key metabolic enzyme in the glycolysis, and facilitated NPC cells proliferation by blocking apoptosis (99). This suggests that the tumor-causing protein LMP1 alters mitochondrial morphology, possibly through the modifications of enzymes related to glycolytic metabolism in NPC cells and then performs metabolic reprogramming to increase chemoresistance.

### Mitochondrial Fusion Favors OXPHOS to Resist Chemotherapy

Increasing evidence indicates an upregulation of OXPHOS in many types of cancer, such as diffuse large B-cell lymphoma and pancreatic cancer, and identifies them as OXPHOS-addictive tumors (100, 101). Studies in pancreatic cancer have shown that subsets of cancer stem cells with elevated metastatic and neoplastic potential are OXPHOS-dependent (102). Fused mitochondria in tumor stem cells are more dependent on OXPHOS because they increase MMP expression, oxygen consumption, and mitochondrial biogenesis (100, 103). Usually, larger mitochondrial networks arising from fusions are observed in metabolically active cells (104). High expression of Mfn2, enhanced OXPHOS respiratory complex and ATP synthase, which facilitates the proliferation and progression of tumor cells, thus inducing doxorubicin resistance in Jurkat leukemia cells (105). In hepatoma cells, highly activated mTOR signaling increases the interaction of the M2 isoform of pyruvate kinase 2 (PKM2) and Mfn2 by phosphorylating Mfn2 and thereby inhibiting the activity of PKM2 and glycolysis. This further suggests that the mTOR/Mfn2/PKM2 signaling axis couples the shift of glycolysis to OXPHOS to promote cancer cell growth (106). The mechanisms probably involves mitochondrial fusion proteins, which would mediate metabolic related-enzymes through multiple signaling pathways to achieve resistance to apoptosis.

**Figure 4:**
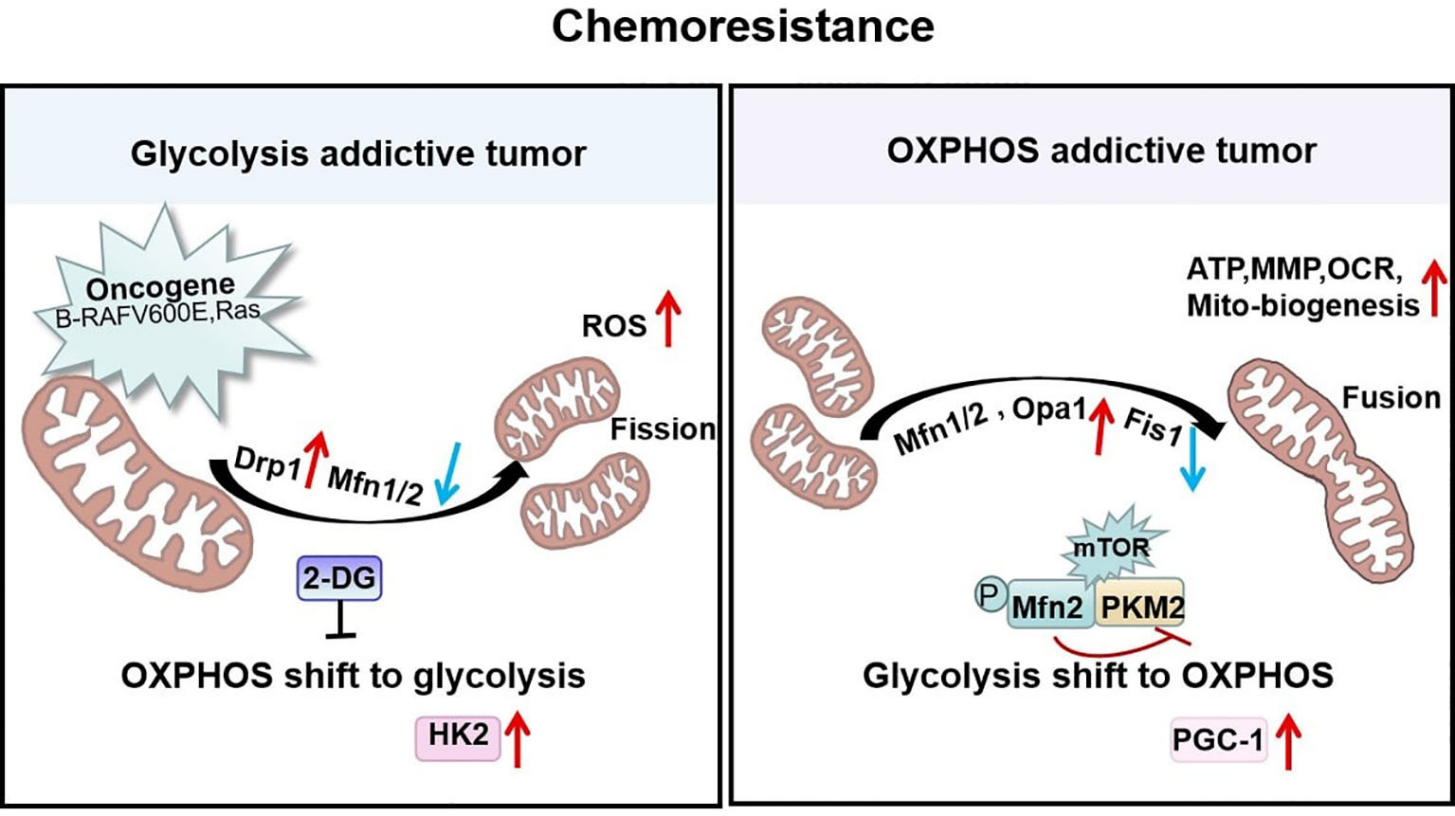
Mitochondrial shaping protein-dependent metabolism and chemoresistance. Tumors addicted to glycolysis exhibit fragmented mitochondria that are predominantly Drp1-dependent with high reactive oxygen species (ROS) levels to enhance chemoresistance. In contrast, tumors addicted to OXPHOS exhibit high Mfn1/2-regulated fused mitochondria possessing elevated ATP, MMP, OCR, and mitochondrial biogenesis to resist chemotherapy.

Consistent with these findings, the mitochondrial characteristics of paclitaxel-resistant lung cancer cells are significantly altered. These changes include decreased mitochondrial volume and membrane potential and a high activation of the mitochondrial biotransformation pathway, with decreased expression of the outer mitochondrial membrane receptor protein Fis1 and increased expression of PGC-1 and the fusion protein Mfn1/2 (107). PGC-1 is a co-regulator that mediates transcription factors for mitochondrial biogenesis and influences mitochondrial respiration, reactive oxygen defenses, and fatty acid metabolism (108). The paclitaxel-resistant cells with high levels of PGC-1 induced mitochondria to form a net-like structure to resist external damage and help mitochondria escape autophagy by increasing the efficiency of ATP synthesis. All of these studies show that the mitochondrial fusion protein Mfn1/2 can stimulate the mitochondrial biogenesis pathway and maintain mitochondrial activity in paclitaxel-resistant cancer cells (107). In addition, with long-term exposure to cisplatin, OPA1-dependent mitochondrial fusion gradually increases. Thus, the expression of PARP-1 and tumor cell apoptosis was decreased leading to cisplatin resistance. At the same time, histone deacetylase Sirt1 was also increased. Sirt1 is an important assessment marker for tumor cell remodeling and the mitochondrial metabolic shift to OXPHOS and resistance to cisplatin treatment (109). This heightened resistance may be attributable to the observation that the activity of SIRT1 is closely controlled by the content of the mitochondrial OXPHOS metabolite NAD^+^. Thus mitochondrial elongation favors its cristae formation and the assembly of respiratory complexes enhancing OXPHOS. This mitochondrial change activates SIRT1 by enhancing NAD^+^ levels, which inhibited glycolysis in response to energy stress and promotes tumor cell survival (110). This study implies that tumor cells respond to chemotherapeutic agents by adjusting mitochondrial morphology and that mitochondrial fusion supports enough energy to lead to the long-term effect of cisplatin-resistant therapy (**Figure 4**).

### Micro RNA Drives Mitochondrial Dynamics and Energy Metabolism to Modulate Chemoresistance

Generally, miR-488 exhibits low expression in chemoresistant cancer cells, which can suppress mitochondrial fission proteins, such as Drp1 and Fis1, by decreasing downstream oncoprotein Six1. As mitochondrial fission reduces, the increased activity of the respiratory chain complex allows for the induction of ROS production and a decrease in MMP. This indicates that dampening the Six1/Drp1 signaling pathway contributes to the suppression of cisplatin resistance (111). In addition, different microRNAs perform various functions. For instance, miR-148a-3p governs cisplatin sensitivity by downregulating A-kinase anchoring protein 1 (AKAP1), which in turn promotes mitochondrial fission. As a substrate of the energy metabolic regulator AMPK, AKAP1 is upregulated in cisplatin-resistant gastric cancer tissues and induces phosphorylation of Drp1 Ser637. The outcome showed inhibition of Drp1 activity and reduced mitochondrial fission, which promoted cell survival leading to increased cisplatin resistance (112). These discoveries implied that microRNA rebuilding of mitochondrial structure can synergize with chemotherapeutic agents to inhibit tumor growth.

## Virus-Driven Drp1-Dependent Mitochondrial Fission Mediates Chemoresistance

Recently, tumor mitochondrial fission conducted by various onco-viruses has been demonstrated. Hepatitis B and C viruses (HBV, HCV) enhance the expression and activity of Drp1 in hepatocellular carcinoma cells, causing an induction of mitochondrial fission and mitophagy that alleviate virus-evoked apoptosis (113, 114). In gastric and breast cancers, the EBV latent membrane protein 2A (LMP2A) stimulates mitochondrial fission and triggers cell migration and EMT (115), suggesting that virus-driven mitochondrial fission boosts tumor cell survival and may be an important mechanism by which viruses mediate resistance to cancer therapy. Our recent study revealed that EBV-LMP1 differentially regulates the signaling axes of AMPK/p-Drp1Ser637 and cyclin B1-Cdk1/p-Drp1Ser616 leading to remodeling of mitochondrial morphology and function. Furthermore, clinical NPC samples indicate that high Drp1 activity is associated with a poor prognosis (98). EBV-LMP1 induced mitochondrial fragmentation, resulting in an increased IC50 value of cisplatin in NPC. Metformin and cucurbitacin E targeting the Drp1 signaling axes enhanced the sensitivity of cisplatin *in vitro* and *in vivo*. This is probably due to the observation that in cisplatin-resistant cancer cells, mitochondria are able to tolerate mtDNA damage and accelerate fission. Importantly, regaining control of mitochondrial mass and maintaining cancer cell survival, suggests an integrated link between mitochondrial dynamics and chemoresistance triggered by tumor virus-stress offers new research directions for cancer therapy.

## Targeted Drugs for Mitochondria-Shaping Proteins

Mitochondria-shaping proteins can be used as cancer therapeutic targets, and their expression and activity can be predictive of tumor development or as prognostic biomarkers. A variety of drugs directly targeting mitochondria-shaping proteins have shown great promise in reducing the viability and proliferation of cancer cells. For instance, Mdivi-1 is a specific Drp1 inhibitor that is currently widely used in tumor research (116). It impairs the oligomerization of Drp1 on OMM and its ability to promote GTP hydrolysis (116). Given the role of Drp1-mediated mitochondrial fission in tumor cell proliferation and metastasis, Mdivi-1 has potential antitumor effects (117). Thus, Mdivi-1 can inhibit cell proliferation by antagonizing the highly activated Drp1 in tumor cells, with potential chemosensitizing functions. However, it has been reported that in neuronal cell lines, mdivi-1 regulates mitochondrial ROS levels and ETC complex I, which was independent of mitochondrial length or Drp1 (118). Another Drp1 inhibitor, p110, blocks the recruitment of Drp1 by Fis1, thereby inhibiting Drp1 translocation to the outer mitochondrial membrane, leading to mitochondrial fragmentation and massive ROS production, which in turn affects apoptosis and cell viability, and is mainly used in neurodegenerative diseases (119). Leflunomide is an FDA-approved drug for the treatment of rheumatoid arthritis, and its pharmacological role is to activate Mfn2-mediated mitochondrial fusion by inhibiting dihydroorotate dehydrogenase (DHODH) (120). In pancreatic cancer cells, leflunomide activates Mfn2, and through inhibition of *de novo* pyrimidine synthesis, prevents tumor cell growth and enhances the chemosensitivity of gemcitabine (121). To date, evidence suggests that specific inhibitors or activators of mitochondrial fission and fusion are not widely used in the clinic. In fact, most studies deal with only non-specific targeting agents, as exemplified by the inhibitor of upstream regulators that have been approved for clinical use. The synergistic interaction of these compounds with chemotherapeutics can also emphasize the multidimensional function of mitochondrial dynamics in cancer chemotherapy (**Table 2**). More relevant, a critical need exists to develop and identify more drugs that directly interfere with mitochondria-shaping proteins.

**Table 2:** Summary of targeted mitochondrial dynamics synergistic chemotherapy.

Cancer types	Chemotherapeutics	Intervention	Mitochondrial shaping proteins	Findings	Refs
Hepatocellular carcinoma	Cisplatin	Drp1 inhibitor/Mdivi-1	Drp1	Mdivi-1 targets mitochondrial autophagy, increasing cisplatin-induced apoptosis	(66)
chollangiocarcinoma	Cisplatin	Bcl2 inhibitor/ABT737	Drp1	ABT737 promotes the ratio of Drp1 60 kD/80 kD form in mitochondria by acting in mitochondrial fission-dependent mitophagy, increasing the sensitivity to cisplatin.	(69)
Ovarian cancers	Cisplatin	Piceatannol	Drp1	Piceatannol to enhance CDDP sensitivity, and it acts on p53, XIAP, and mitochondrial fission, leading to more effective induction of apoptosis.	(76)
Ovarian cancers	Cisplatin	ROS inhibitor /Piperlongumine	Drp1	Piperlongumine-induced apoptosis appeared to be mediated by Drp1-dependent mitochondrial fission.	(77)
Breast cancer	Doxorubicin	Catehol	Drp1	Liensinine inhibiting autophagy, activates Drp1 to increase apoptosis and improve chemosensitivity.	(70)
Nasopharyngeal cancer	5-fluorouracil	Resveratrol	Drp1	RSV inhibits the Cox-2/p53/Drp1 signaling axis to inhibit mitochondrial fission, increased sensitivity of NPC to 5-FU.	(41)
Ovarian cancers	Cisplatin	OMA1	OPA1	P53 activates Oma1, promoting OPA1 by cleavage L-OPA1 to S-OPA1, which reduces miyochondrial fusion and sensitizes to cisplatin.	(78)
Breast cancer	Cisplatin	Drp1 inhibitor/Mdivi-1	Drp1	Mdivi-1 target Drp1to increase MMP, the generation of ROS and apoptosis.	(81)
Ovarian cancers	Cisplatin	Antioxidant	Drp1/Mfn 1	hypoxia-induced ROS triggers mitochondrial fission by down-regulating p-Drp1 (Ser637) and Mfn1 expression levels in ovarian cancer cells, thereby inducing cisplatin resistance	(83)
lymphoblastic leukemia	Cisplatin	MAPK/ERK inhibitor, PD325901	Drp1	ERK triggers Drp1-dependent mitochondrial fragmentation, leading to a decrease in mitochondrial ROS levels and a promotion in glycolytic phenotypic switch that resulting in chemoresistance	(87)
Nasopharyngeal cancer	Cisplatin	Metformin/Cucurbitacin E	Drp1	Decreases mitochondrial fission and increases chemotherapy sensitivity to cisplatin	(98)
Neuroblastoma	Etoposide and doxorubicin	Glycolysis inhibitors /2-DG	Drp1	2-DG suppresses mitochondrial fission and increases the pro-apoptotic protein BCL2L11/BimBCL2L11/Bim and attenuate the anti-apoptotic effect of survivin, making the tumor sensitive to chemotherapeutic agent.	(97)
Neuroblastoma	Cisplatin	Targeting Sirt1	OPA1	Sirt1 remodeling mitochondrial metabolic to OXPHOS shift resistance to cisplatin treatment.	(109)
Lung cancer	Paclitaxel	Targeting PGC1, Mfn1/2	Mfn1/2	PGC1 induced Mfn1/2 dependent mitochondrial fusion to resist paclitaxel and help mitochondria escape autophagy by increasing the efficiency of ATP synthesis.	(107)
Ovarian cancer	Cisplatin	Restoration of miR-488	Drp1	Inhibition of the Six1/Drp1 signaling pathway contributes to the suppression of cisplatin resistance.	(111)
Gastric cancer	Cisplatin	Reconstitution of miR-148a-3p	Drp1	Downregulation of KAP1, which in turn promotes mitochondrial fission.	(112)

## Conclusion

Lately, mitochondrial dynamics has been extensively applied in classifying tumors, predicting clinical prognosis, and assessing therapeutic response. The study of delicate modifications of mitochondrial fission and fusion in relation to tumor development appears to be an active academic frontier. The chemoresistant phenotype of tumor cells may result from alterations in mitochondrial dynamics proteins and their signaling pathways, affecting tumor cell death and metabolic changes.

Disturbed energy metabolism is an increasingly recognized mechanism by which mitochondria-shaping proteins mediate chemoresistance. Tumors addicted to glycolysis show predominantly Drp1-dependent mitochondrial fragmentation in order to enhance chemoresistance. In contrast, tumors addicted to OXPHOS exhibit high Mfn1/2-regulated fused mitochondria to resist chemotherapy. On the basis of metabolic typing, Drp1 or Mfn1/2 proteins might serve as markers for further stratification of tumor chemotherapy resistance. Furthermore, our recent findings reveal that the onco-virus protein EBV-LMP1 exerts a signaling function that indirectly regulates mitochondrial shaping protein-induced chemoresistance. Other tumor causing viruses, such as HBV and HCV have been reported to drive mitochondrial fission by activating Drp1 to evade apoptosis. The biological effects may affect chemotherapy sensitivity of HBV- or HCV-related liver cancer, but the mechanism is still unclear. Of course, these viruses may play an indirect regulatory role similar to EBV viruses or encoded proteins might translocate to the mitochondria and interact with mitochondria-shaping proteins directly, which needs to be further investigated.

Overall, we reviewed the alterations of mitochondrial dynamics in cancer chemotherapy from a tumor cell perspective. Moreover, its role in the tumor microenvironment should also be brought to our attention. Mitochondria have essential functions in both innate and adaptive immunity. Mitochondrial remodeling allows quiescent immune cells to rapidly change their metabolism and become activated, producing mediators, such as cytokines, chemokines and even metabolites to execute an effective immune response (122).

In innate immunity, mitochondrial fusion enhances the formation of (extra-neutrophilic traps) NETs in neutrophils (123) and promotes M2-like polarization of bone marrow-derived macrophages (124). Furthermore, hypoxia invokes excessive mitochondrial fission in liver tumor-infiltrating NK cells, while enhancing mTOR-Drp1 signaling and decreasing the anti-tumor activity of NK cells (125). In adaptive immunity, mitochondria-shaping proteins are required for T cell activation, differentiation, migration (126). OPA1-regulated T cell mitochondrial fusion promotes T memory cell metabolism by altering mitochondrial cristae, leading to activation of the ETC complex and efficient OXPHOS, improving cellular immunotherapy against tumors (127).

The main effect of chemotherapeutic drugs in killing tumor cells is non-immunotoxicity dependent, but there is some immune activating or inhibiting activity (128). Based on this, some questions need to be further explored, whether chemotherapeutic drugs act on mitochondria-shaping proteins of immune cells and ultimately affect their anti-tumor immune response. Whether targeting mitochondria-shaping proteins to regulate mitochondrial morphology in tumor cells also affects the dynamics of mitochondria in immune cells. A dual role in the development of tumor chemotherapy by intervening in mitochondrial dynamics, which is able to both accelerate the death of tumor cells and enhance the anti-tumor effect of immune cells. Therefore, a comprehensive understanding of the role of mitochondrial dynamics in cancer chemotherapy cannot ignore the activity of the immune system.

## Author Contributions

LX and YC conceived and designed the study. LX and YC participated in its drafting. AB helped to edit the manuscript. TZ and YX performed the visualization. All authors contributed to the article and approved the submitted version.

## Funding

This study was supported by the National Natural Science Foundation of China (82103019, 81430064, and 81602402).

## Conflict of Interest

The authors declare that the research was conducted in the absence of any commercial or financial relationships that could be construed as a potential conflict of interest.

## Publisher’s Note

All claims expressed in this article are solely those of the authors and do not necessarily represent those of their affiliated organizations, or those of the publisher, the editors and the reviewers. Any product that may be evaluated in this article, or claim that may be made by its manufacturer, is not guaranteed or endorsed by the publisher.

## Abbreviations

Drp, Mitochondrial dynamin-related protein; Mff, Mitochondrial fission factor; MiD49/51, Mitochondrial dynamics protein of 49/51kDa; Mfn1/2, Mitofusin1/2; OPA1, Optic atrophy 1; HCC, Hepatocellular carcinoma cells; NPC, Nasopharyngeal cancer cells; RSV, Resveratrol 5-FU, 5-fluorouracil; XIAP, X-linked inhibitor of apoptosis protein; MMP, Mitochondrial membrane potential; ERKs, Extracellular signal-regulated kinases; HBV, HCV, Hepatitis B and C viruses; EMT, Epithelial-mesenchymal transition; LMP1, EBV encodes oncoprotein latent membrane protein 1; BTICs, Brain tumor initiating cells; OXPHOS, Oxidative phosphorylation; 2-DG, 2-deoxy-Dglucose; PKM2, pyruvate kinase; AKAP1, A-kinase anchoring protein 1.
